# Physiological and Morphological Implications of Using Composts with Different Compositions in the Production of Cucumber Seedlings

**DOI:** 10.3390/ijms241814400

**Published:** 2023-09-21

**Authors:** Anita Zapałowska, Natalia Matłok, Tomasz Piechowiak, Małgorzata Szostek, Czesław Puchalski, Maciej Balawejder

**Affiliations:** 1 Department of Agriculture and Waste Management, Collegium of Natural Sciences, University of Rzeszów, St. Ćwiklinskiej 1a, 35-601 Rzeszów, Poland; 2 Department of Food and Agriculture Production Engineering, Collegium of Natural Sciences, University of Rzeszów, St. Zelwerowicza 4, 35-601 Rzeszów, Poland; nmatlok@ur.edu.pl; 3 Department of Chemistry and Food Toxicology, Collegium of Natural Sciences, University of Rzeszów, St. Ćwiklińskiej 1a, 35-601 Rzeszów, Poland; tpiechowiak@ur.edu.pl (T.P.); mbalawejder@ur.edu.pl (M.B.); 4 Department of Soil Science Environmental Chemistry and Hydrology, Collegium of Natural Sciences, University of Rzeszów, St. Zelwerowicza 8b, 35-601 Rzeszów, Poland; mszostek@ur.edu.pl; 5 Department of Bioenergetics, Food Analysis and Microbiology, Institute of Food Technology and Nutrition, Collegium of Natural Sciences, University of Rzeszów, St. Ćwiklińskiej 2D, 35-601 Rzeszów, Poland; cpuchlaski@ur.edu.pl

**Keywords:** sewage sludge, horticultural substrates, *Cucumis sativus* L., chlorophyll content, chlorophyll fluorescence, plant biometrics

## Abstract

Compost has a broad application in terms of the improvement of the soil properties. This research work was conducted to present the molecular implications of using compost obtained from different substrates to improve soil parameters for cucumber seedlings cultivation. In the experiment, the following compost mixtures were used: sewage sludge (80%) + sawdust (20%); sewage sludge (40%) + sawdust (10%) + biodegradable garden and park waste (50%); biodegradable garden and park waste (90%) + sawdust (10%); sewage sludge (80%) + sawdust (20%) + *Eisenia fetida*; sewage sludge (40%) + sawdust (10%) + biodegradable garden and park waste (50%) + *Eisenia fetida*; biodegradable garden and park waste (90%) + sawdust (10%) + *Eisenia fetida*. The final substrate compositions consisted of compost mixtures and deacidified peat(O) (pH 6.97; Corg content—55%, N content—2.3%), serving as a structural additive, in different mass ratios (mass %). The produced plants underwent biometric and physiological measurements as well as enzymatic analyses of stress markers. Based on the conducted studies, it has been found that the substrate productivity depends not only on the content of nutrient components but also on their structure, which is moderated by the proportion of peat in the substrate. The most effective and promising substrate for cucumber seedling production was variant 2 (I), which consisted of 25% compost from sewage sludge (40%) + sawdust (10%) + biodegradable garden and park waste (50%) and 75% deacidified peat. Despite the richness of the other substrates, inferior parameters of the produced seedlings were observed. The analysis of the enzymatic activity of stress markers showed that these substrates caused stress in the plants produced. The study’s results showed that this stress was caused by the presence of *Eisenia fetida*, which damaged the developing root system of plants in the limited volume of substrate (production containers). The adverse influence of *Eisenia fetida* on the plants produced could possibly be eliminated by thermal treatment of the compost, although this could lead to significant changes in composition.

## 1. Introduction

Seed cucumber (*Cucumis sativus* L.) is a widespread horticultural plant worldwide due to its economic and social significance [1]. The production of healthy seed cucumber seedlings is a key factor that determines the quantity and quality of subsequent yields. Traditional seedling production methods are expensive and require the use of substrates, mainly peat-based, the extraction of which can lead to negative environmental impacts [2].

In recent years, there has been an increased interest in seeking innovative solutions for the production of horticultural plant seedlings that simultaneously minimize negative environmental impacts and enhance the efficiency of producing seedlings with desired quality parameters. One such solution is the use of composts as substrates for seedling production [3]. Composts are the product of organic decomposition and serve as a valuable source of nutrients for plants [4]. Their application as an additive to peat-based substrates can contribute to improving substrate quality, increasing plant productivity, and reducing the need for mineral fertilizers. Furthermore, due to their significant organic matter content, composts can also enhance the soil structure and increase its retention capabilities [2,5].

Another waste material serving as an alternative source of nutrient compounds for plants is stabilized sewage sludge. The agricultural and horticultural utilization of sewage sludge for plant fertilization is an alternative method for their disposal and utilization [6,7]. The nutrient-rich composition of sewage sludge, primarily nitrogen and phosphorus, positively influences the growth and development of various crop species. However, despite their agricultural usefulness, their application might be restricted by the presence of heavy metals and pharmaceuticals [8,9], which is regulated by the EU directive on sewage sludge, defining maximum limits for six metals in sludge [10].

Peat is the main component of seedlings’ growing media. Advantages of using peat-based substrates depend on their favorable physical characteristics. They have a relatively low bulk density, a high porosity and a high nutrient exchange capacity, providing an optimal raw material in which the desired nutrient contents and pH can easily be adjusted. Peat-based substrates thus provide an ideal basis for plant propagation and growth. They improve plant growth by supplying nutrients, stimulating growth, enhancing flowering and fruiting, increasing the number of beneficial microbes and controlling pests and diseases. As shown in numerous studies, the replacement of nursery substrates for conventional cultivation with composted agricultural waste has achieved good results [11,12,13].

The aim of this study was to determine the impact of using innovative compost with varying organic matter compositions on selected physiological and biochemical parameters of cucumber seedlings (*Cucumis sativus* L.) using advanced molecular methods.

It was hypothesized that compost derived from the mixed biowastes with the combination of deacidified peat affects the growth of vegetable seedlings. Therefore, the development of the composition of the mixture combination for cucumber cultivation (*Cucumis sativus* L.) was tested.

## 2. Results and Discussion

### 2.1. Characteristics of Physical and Chemical Properties of Substrates

The substrates employed in the study exhibited diverse physicochemical properties, depending on the type of compost used (Table 1). The deacidified peat utilized in the experiment displayed a neutral pH (pH = 6.97) and low salinity (EC = 136 µS cm^−1^). The average content of Total Organic Carbon (TOC) and Total Nitrogen (N_total_) in the peat was 55% and 2.30%, respectively, with a C:N ratio of 23.9. The average content of available forms of phosphorus (P) and potassium (K) was 46.9 and 33.0 mg 100 g^−1^, while the overall content of magnesium (Mg) and calcium (Ca) was 1.37 and 2.13 g kg^−1^. The properties of the substrates used in the experiment varied depending on the compost variant and its contribution to the resulting substrate. The determined pH values of the produced substrates ranged from 5.90 to 7.87. The lowest values were observed for substrate 4 (III), whereas the highest were noted for substrate 6 (III) (Table 1). Meanwhile, for variant 4 (III), the highest EC values were recorded, averaging 1958 µS cm^−1^. The participation of specific composts in the resulting substrates also influenced the TOC content, N content, and C:N ratio. Among the analyzed variants, substrates 1 and 4 exhibited the highest proportion of TOC, where, depending on the addition of peat, the average proportion of this component ranged from 52.1% to 46.2% and from 51.8% to 45.4%, respectively. The lowest TOC content, however, was observed in the case of substrate 3. Similar dependencies were also noted for N content. Substrate 3 also featured the lowest average content of P_2_O_5_, where, depending on the addition of peat, the average content of this element ranged from 50 to 151 mg kg^−1^. Conversely, the highest content of this element was observed in substrate 1 (average 325–975 mg kg^−1^). The content of K_2_O, on the other hand, was highest in substrates 2, 5, and 6, while Mg and Ca content were highest in substrate 6 (Table 1).

The content of microelements was also analyzed in the substrates produced in the experiment (Table 2). The average content of these elements in the composts used in the experiment fell within the limits set in the legal regulations [10,14,15]

The deacidified peat used in the experiment exhibited low levels of Pb, Cr, Cu, Ni, Cd, Zn, and Hg. In contrast, the use of composts in some cases significantly influenced the increase in the levels of these elements in the substrates used in the experiment. Among the analyzed variants, the substrate with the addition of compost 6 and peat exhibited the highest content of Pb and Cr. Meanwhile, in the substrate with the addition of compost 4, the highest levels of Zn, Cu, Cd, and Hg were observed compared to the other experiment variants. Additionally, the substrate with the addition of compost 3 recorded the highest average content of Ni compared to the other variants.

### 2.2. Biometric Parameters of Plants

**Figure 1:**
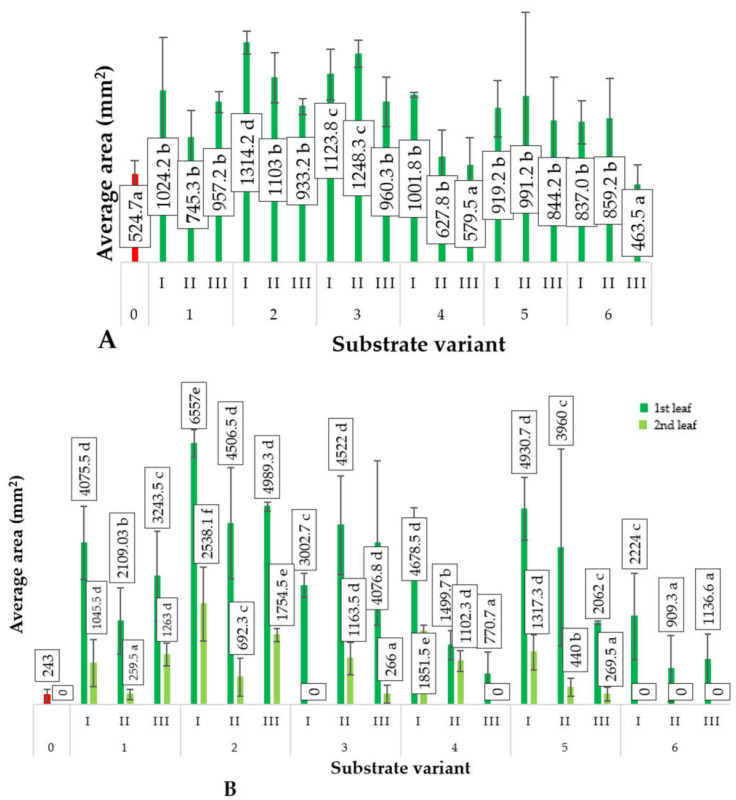
Surface area of cotyledons (**A**) and leaves (**B**) of ground cucumber plants (*n* = 20) depending on the applied substrate on the 21st day after seed sowing. Differences in the results between substrates; significant differences at the *p* < 0.05 level; different lowercase letters indicate significant differences between substrate variants. Substrate variants: 0, 1, 2, 3, 4, 5, 6. Compost variants: I (25% compost + 75% peat), II (50% compost + 50% peat), III (75% compost + 25% peat).

**Figure 2:**
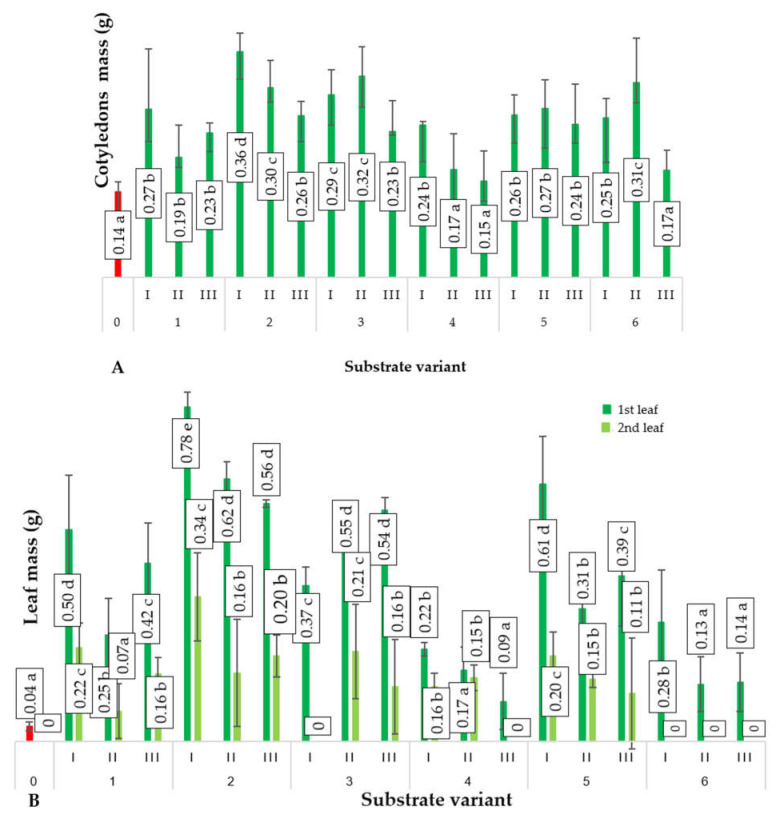
Average values of mass of cotyledons (**A**) and leaves (**B**) of ground cucumber plants (*n* = 20) depending on the substrate on the 21st day after seed sowing. Differences in the results between the substrate; difference at significant level *p* < 0.05; different lowercase letters indicate significant differences between substrate variants. Substrate variants: 0, 1, 2, 3, 4, 5, 6. Compost variants: I (25% compost + 75% peat), II (50% compost + 50% peat), III (75% compost + 25% peat).

The produced substrates significantly influenced the growth of cucumber plants during the initial stages of development. Cotyledons, which emerge from the developing embryo to initiate growth and development, mainly rely on stored materials within the seeds [16]. However, the applied substrates affected their surface area and mass after 3 weeks of sowing (Figure 1 and Figure 2). The most favorable biometric parameters were exhibited by the cotyledons of cucumber seedlings produced using substrate 2(I). The mass of these cotyledons was 157% higher compared to the control variant. Additionally, the surface area of cucumber cotyledons cultivated on substrate 2(I) was 250% higher than that of the control variant. Despite containing only 25% compost B additive, this substrate resulted in the best plant growth. This suggests that the growth and mass of cucumber cotyledons were not directly regulated by the content of biogens in the tested substrates but rather by their structure.

A similar trend was observed regarding the influence of the employed substrates for producing cucumber seedlings on the surface area as well as the number and mass of leaves (Figure 2). The development of leaves in plants is influenced by two key factors: substrate richness and the bioavailability of plant nutrients. The bioavailability of essential nutrients for plants depends on its physical characteristics, primarily its structure, which is determined by bulk density. Visual assessment of the produced composts indicates their dense, cohesive structure. Such physical properties hinder the growth of the newly forming root systems of germinating plants. Nevertheless, their chemical composition (Table 1) indicates richness in the micro- and macronutrients of the nutritional value for plants along with permissible levels of other components (e.g., toxic metals). Furthermore, these components exist in various proportions depending on the components used in their production, allowing the preparation of substrates with diverse compositions and the ability to select them for the effectiveness of producing horticultural plant seedlings, including ground cucumber. It is important to note that these substrates are produced using processed waste materials, leading to reduced environmental impact resulting from human activities, including agricultural practices.

The alteration of substrate structures made from composts was achieved by employing a structural additive. One of the most commonly used organic materials in horticultural production is peat [17], which was added in various mass proportions to the produced substrates. This addition allowed the modification of bulk density, which influences several physical properties of the substrate, such as porosity, air permeability, water retention, and the potential for root system development, which determines the availability of nutrients for plants. For variants 4–6, analogous composts to those in variants 1–3 were used, but they were produced through vermicomposting using *Eisenia fetida*.

**Figure 3:**
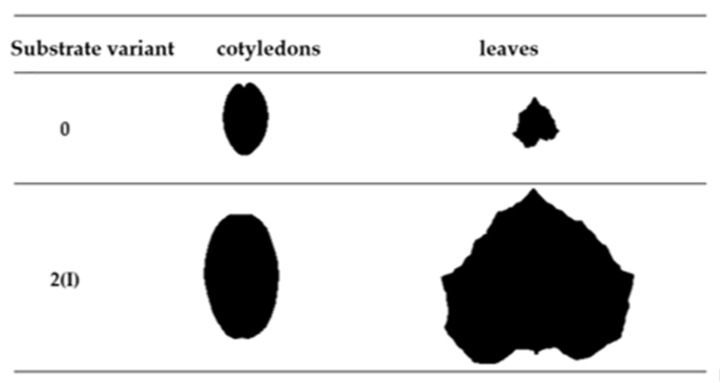
Examples of cotyledons surface area and leaves of seedling cucumbers produced on control substrate (0) and substrate 2(I) on the 21st day after seed sowing. Scale (1:3).

**Figure 4:**
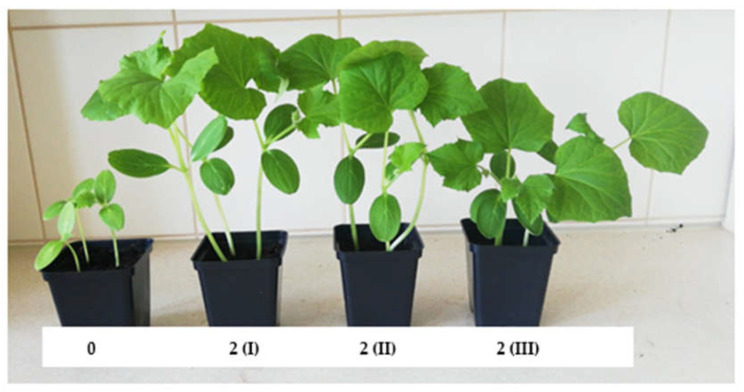
Growth habit of seedling cucumber plants produced on control substrate (0) and substrate 2 (I), (II), (III) on the 21st day after seed sowing.

**Figure 5:**
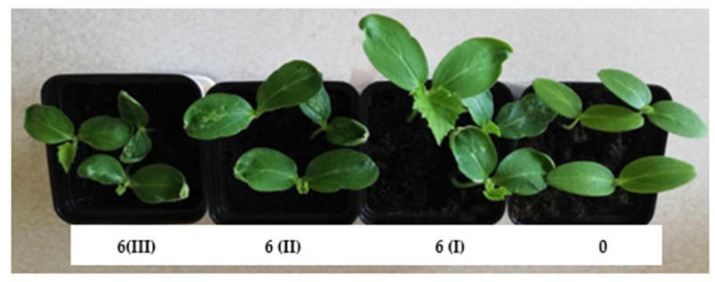
Deformations of cotyledons and leaves of seedling cucumber plants produced on substrate 6 (III), (II), (I) compared to control (0) on the 10th day after seed sowing.

The most effective substrate (variant 2(I)) for producing ground cucumber seedlings contained 25% compost (derived from sewage sludge (40%), sawdust (10%), and organic waste (50%)) and 75% deacidified peat. The use of this substrate led to the production of ground cucumber seedlings with the best biometric parameters (Figure 1, Figure 2, Figure 3 and Figure 4). Ground cucumber plants grown on substrate 2(I) developed second leaves with an average mass of 0.78 g (first leaf) and 0.34 g (second leaf). The surface area of these leaves averaged 6557.0 mm^2^ (first leaf) and 2538.1 mm^2^ (second leaf). Notably, the plants from the control group during the same period (21 days after sowing) produced only 1 leaf, with a mass and surface area 35.9% and 2600% smaller, respectively, than the values of these parameters for the plants in variant 2(I) (Figure 1 and Figure 2). This substrate exhibited one of the lowest bulk densities alongside a favorable nutritional composition (Table 1). The increase in bulk density due to the higher compost content in substrate 2 led to lower biometric parameters of ground cucumber plants despite the higher nutrient content in these substrates (variants 2(II) and 2(III)). For the other substrates (variants 5–6), where the compost content was also 25%, the registered parameters of cotyledons and leaves of ground cucumber plants were considerably lower. The observed plant morphology indicated the influence of other stress-inducing factors, which were manifested by the deformation of cotyledons and leaves (Figure 5). Furthermore, a visual assessment of the isolated root systems of ground cucumber plants produced on these substrates (variants 4–6) indicated mechanical damage due to the feeding of soil organisms. In the vicinity of these roots, individuals of *Eisenia fetida* were observed at various stages of development, which likely contributed to the damage of root systems and consequently restricted plant growth. This finding is surprising in light of the available literature, which suggests that vermicomposting significantly improves the structure of produced composts [18,19,20]. However, in the limited volume of production containers for horticultural plant seedlings, an excessive increase in *Eisenia fetida* biomass occurred. These organisms, in search of food, fed on the produced ground cucumber plants. This phenomenon was confirmed by other researchers [21]. They found that with increasing compost content in the substrate, the negative impact of earthworms contained in the compost on plant growth and development becomes worse. Many authors assert that the influence of earthworms contained in compost on plant growth depends on various factors, including the duration of the plant’s vegetative period. Plants with longer vegetative periods cope better with the undesirable attack of nematodes by earthworms [21,22].

### 2.3. Relative Chlorophyll Content (SPAD)

**Figure 6:**
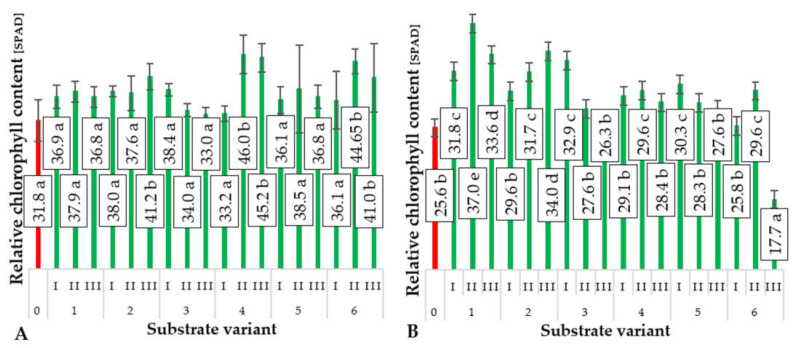
The relative content of chlorophyll (SPAD) in cucumber cotyledons (**A**) and leaves (**B**) depending on the type of substrate (*n* = 20). Differences in the results between the substrate; difference at significant level *p* < 0.05; different lowercase letters indicate significant differences between substrate variants. Substrate variants: 0, 1, 2, 3, 4, 5, 6. Compost variants: I (25% compost + 75% peat), II (50% compost + 50% peat), III (75% compost + 25% peat).

An important physiological parameter of plants that indicates their nitrogen status is the relative chlorophyll content in plant leaves [23,24]. Substrates characterized by the highest total nitrogen content were those produced with the involvement of compost A, which was made from 80% sewage sludge and 20% sawdust (Table 1). Probably the elevated total nitrogen content in compost A translated into a higher relative chlorophyll content in the biomass of the produced ground cucumber plants. However, this relationship was not linear and did not directly depend on the nitrogen content in the final substrates but rather on their physical properties. The highest relative chlorophyll content in the leaves of cucumber plants (Figure 6B) was observed in the case of substrate 1(II), which was composed of 50% compost and 50% deacidified peat. Increasing the proportion of compost, despite the higher nitrogen content in the resultant substrate, led to a decrease in the relative chlorophyll content. This was likely due to the limited nutrient availability, primarily nitrogen, because of the structure of that substrate. In the case of the most productive substrate (Variant 2(I)), which used compost with a lower nitrogen content compared to substrate 1(II), increasing the proportion of compost in the substrate resulted in a linear increase in the relative chlorophyll content in the leaves of the ground cucumber plants. Analysis of the obtained results indicated that the relative chlorophyll content in the leaves of cucumber plants strongly depends on the nitrogen content in the substrates on which the plants were grown. This is confirmed by the Pearson linear correlation coefficient, which is 0.998. High chlorophyll concentration in the leaves of cultivated cucumber plants is closely related to the determined leaf mass, which is inversely proportional to the nitrogen concentration in the substrate (Pearson coefficient r = −0.9672). This is likely due to the influence of other growth-limiting factors related to the physical properties of the substrates, especially bulk density. For cucumber seedlings produced on substrates made with composts C, D, E, and F (substrate variants 4–6), lower chlorophyll contents were observed in the leaves (Figure 6B), but in most cases, these were higher than in the control plants. The lower relative chlorophyll content in the leaves of plants grown on these substrates indicates a less favorable combination of physical and chemical substrate parameters compared to substrates involving composts A and B or other limiting factors. The likely factor responsible for this in the case of substrates marked by variants 4–6 is the presence of soil organisms, mainly *Eisenia fetida*, which is used in the vermicomposting process.

### 2.4. Chlorophyll Fluorescence Parameters

**Figure 7:**
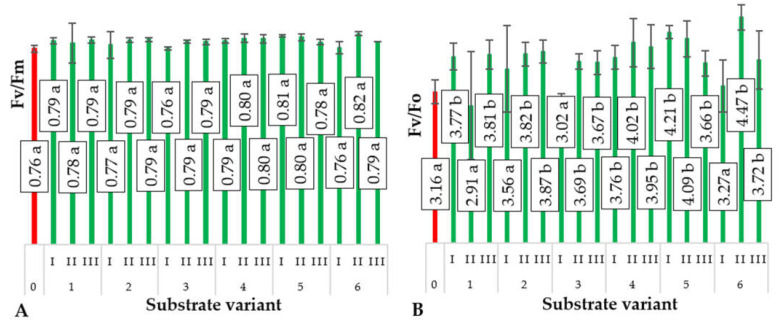
Maximal photochemical efficiency of PSII (F_v_/F_m_) (**A**), average values of chlorophyll fluorescence parameters (maximum quantum yield of primary photochemistry (F_v_/F_o_)) (**B**), in cucumber cotyledons depending on the type of substrate (*n* = 20). Differences in the results between the substrate; difference at significant level *p* < 0.05; different lowercase letters indicate significant differences between substrate variants. Substrate variants: 0, 1, 2, 3, 4, 5, 6. Compost variants: I (25% compost + 75% peat), II (50% compost + 50% peat), III (75% compost + 25% peat).

The relative chlorophyll content in the aboveground biomass of plants serves as an indicator of their photosynthetic capacity [25,26]. However, the efficiency of this process depends on several other factors, such as nutrient and water availability, CO_2_ supply, light availability, and the presence of biotic and abiotic stress factors [27,28]. Instrumental measurement of chlorophyll fluorescence parameters—maximal photochemical efficiency of PSII (Fv/Fm) (Figure 7A) and maximum quantum yield of primary photochemistry (Fv/Fo) (Figure 7B)—allows the determination of the efficiency of this process, particularly under the influence of various environmental factors, including stressors [29,30,31].

Approximately 70% to 90% of the incoming solar radiation is absorbed by the leaf, with around 40% to 60% of this radiation being absorbed by the energy antennae, transferring energy to the PSII reaction centers. Under normal conditions without stress, most of the energy is used in photochemical reactions with only a small fraction being emitted as fluorescence or heat. In the presence of stress, the PSII reaction changes, altering the balance between PSII and PSI and the amount of energy emitted as heat and fluorescence [32]. The substrates used for producing ground cucumber plants exhibited different physical properties and chemical compositions, which were the sole factors differentiating the conditions of plant production and could influence chlorophyll fluorescence parameters, mainly the Fv/Fm ratio, serving as a test for various types of stress affecting PSII [32]. Michałek et al. [33] demonstrated that the application of various factors, including biostimulants, significantly affects the photosynthetic efficiency measured through chlorophyll fluorescence in bean plant production.

**Figure 8:**
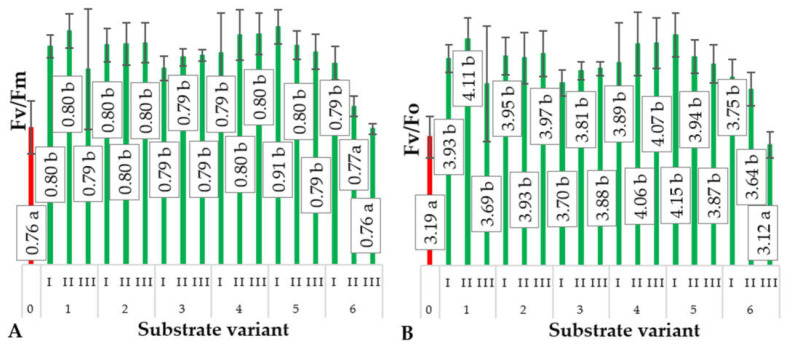
Maximal photochemical efficiency of PSII (F_v_/F_m_) (**A**), average values of chlorophyll fluorescence parameters (maximum quantum yield of primary photochemistry (F_v_/F_o_)) (**B**), in cucumber leaves depending on the type of substrate (*n* = 20). Differences in the results between the substrate; difference at significant level *p* < 0.05; different lowercase letters indicate significant differences between substrate variants. Substrate variants: 0, 1, 2, 3, 4, 5, 6. Compost variants: I (25% compost + 75% peat), II (50% compost + 50% peat), III (75% compost + 25% peat).

Analysis of the obtained results indicates that the lowest maximal photochemical efficiency of PSII (Fv/Fm) was recorded for the leaves of ground cucumber seedlings (Figure 8A) produced on the control substrate and substrate variant 6. Significant differences were observed in the remaining variants. A similar trend was observed for cotyledons. The results of chlorophyll fluorescence measurements did not unequivocally confirm the occurrence of stress induced by the feeding of *Eisenia fetida*, prompting the expansion of the study to include the analysis of biochemical stress markers. Kooten and Snel [34] suggest that non-invasive measurements of chlorophyll fluorescence are not always sufficient markers of plant stress, and their greatest advantage lies in their non-destructive nature for plants.

### 2.5. Antioxidant Enzymes Activity

Enzyme activity measurements: catalases CAT, guaiacol peroxidase GPOX, and superoxide dismutase SOD can serve as markers of plant stress occurring during photosynthesis, one of the products of which is oxygen [35]. This oxygen can transform into reactive oxygen species (ROS) within cellular systems, most of which exhibit high phytotoxicity [36]. In response to oxidative stress, plants alter the activity of enzymes involved in scavenging ROS, including catalases (CATs), guaiacol peroxidase (GPOXs), and superoxide dismutase (SOD).

**Figure 9:**
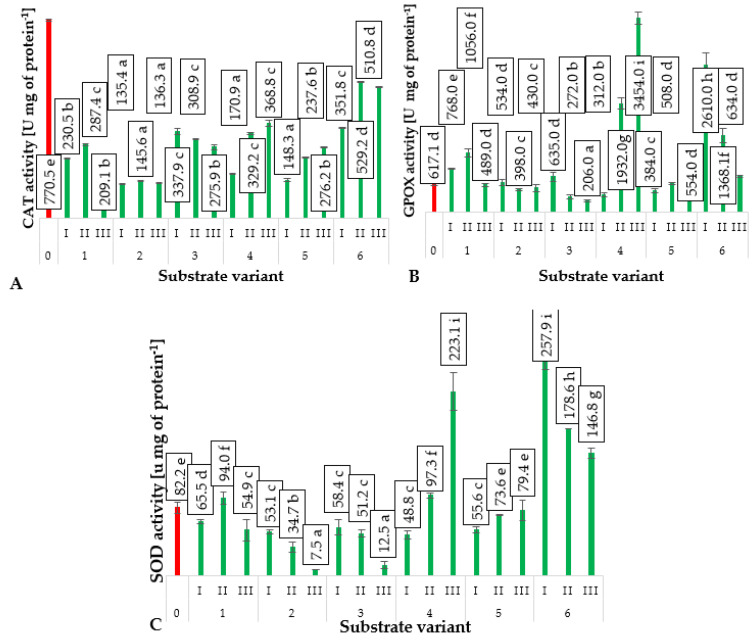
Catalases (CATs) (**A**), guaiacol peroxidase (GPOX) (**B**) and superoxide dismutase (SOD) (**C**) in cucumber leaves depending on the substrate (*n* = 20). Differences in the results between the substrates; difference at significant level *p* < 0.05; different lowercase letters indicate significant differences between substrate variants. Substrate variants: 0, 1, 2, 3, 4, 5, 6. Compost variants: I (25% compost + 75% peat), II (50% compost + 50% peat), III (75% compost + 25% peat).

As demonstrated by Matłok et al. [26], the occurrence of a stress factor significantly modifies the activity of CAT, SOD, and GPOX enzymes. In the case of exposure to ozone [28], it has been shown that ROS are formed within cellular systems, eliciting a response from plants. However, other factors, such as fertilization or the presence of herbivore feeding, activate peroxisomes to produce hydrogen peroxide, which is subsequently scavenged under physiological conditions by CAT and GPOX. This suggests that the highest CAT activity in ground cucumber plants grown on the control substrate (Figure 9A) resulted from the substantial presence of hydrogen peroxide in plant metabolism. The presence of hydrogen peroxide is an important regulatory factor that enables plants to respond to environmental stress by activating defense mechanisms, as demonstrated in studies by Slesak et al. [37]. The highest CAT activity in ground cucumber plants grown on the control substrate was likely due to the necessity of breaking down excess hydrogen peroxide produced in plants due to nutrient deficiencies [38]. The control substrate (Variant O) consisted solely of deacidified peat, which had minimal nutrient content (Table 1). Increased CAT activity was also observed in ground cucumber plants grown on substrates with the addition of compost produced using *Eisenia fetida*, particularly in substrate variant 6, which was additionally the least nutrient rich. The impact of this compost is evidenced by the observed increase in CAT activity with the increasing proportion of this compost in the substrate composition (Figure 9A).

Guaiacol peroxidase (GPOX) is also an enzyme involved in hydrogen peroxide decomposition [39]. However, its activity is a better marker of stress occurrence in plants induced by herbivore feeding, which triggers an overproduction of hydrogen peroxide [40]. Increased GPOX activity was observed in ground cucumber seedlings produced on substrates with the addition of vermicomposts (substrate variants 4–6) (Figure 9B). The activity is not directly correlated with the proportion of compost in the substrate and is the outcome of other factors influencing the antioxidant status of plants. The precursor of hydrogen peroxide formed during photosynthetic metabolism is the superoxide anion radical. This reactive species is quickly converted into hydrogen peroxide under the influence of SOD [41]. Analysis of the results obtained showed that the SOD activity in ground cucumber plants has a high activity in the control group (substrate variant O), and it is also relatively high in the plant groups produced on substrates containing vermicomposts (substrate variants 4–6) (Figure 9C). The high activity of this enzyme in these plants may suggest that SOD activity could be a good marker of stress induced by the feeding of soil organisms on the root system, mainly *Eisenia fetida.*

## 3. Materials and Methods

### 3.1. Seed Material

In the experiment, seeds of the cucumber hybrid variety ‘Śremski’ (PlantiCo, Zielonki, Poland) were used as the seed material. This variety is a popular early cucumber variety known for its high fruit yield and characteristics that make it suitable for processing.

### 3.2. Substrate Preparation Procedure

Compost was prepared using different organic matter in various mass proportions (% by mass):

A. Sewage sludge (80%) + Sawdust (20%);
B. Sewage sludge (40%) + Sawdust (10%) + Biodegradable garden and park waste (50%);
C. Biodegradable garden and park waste (90%) + Sawdust (10%);
D. Sewage sludge (80%) + Sawdust (20%) + *Eisenia fetida*;
E. Sewage sludge (40%) + Sawdust10%) + Biodegradable garden and park waste (50%) + *Eisenia fetida;*
F. Biodegradable garden and park waste (90%) + Sawdust (10%) + *Eisenia fetida.*

The organic mixture was subjected to the controlled composting process in six 1 m^3^ plastic containers for 3 months. The moisture of the organic matter under composting was monitored weekly. Temperature measurements confirmed the existence of four characteristic phases of composting process including mesophilic, thermophilic, cooling and maturation phases.

Based on previously prepared compost and deacidified peat (O) (pH 6.9; Corg content—55%, N content—2.3%) in various mass proportions (% by mass), substrates for cucumber seedling production were prepared as follows:

1. (I): A (75%) + O(25%); (II): A (50%) + O (50%); (III): A(25%) + O (75%);
2. (I): B (75%) + O(25%); (II): B (50%) + O (50%); (III): B(25%) + O (75%);
3. (I): C (75%) + O(25%); (II): C (50%) + O (50%); (III): C(25%) + O (75%);
4. (I): D (75%) + O(25%); (II): D (50%) + O (50%); (III): D(25%) + O (75%);
5. (I): E (75%) + O(25%); (II): E (50%) + O (50%); (III): E(25%) + O (75%);
6. (I): F (75%) + O(25%); (II): F (50%) + O (50%); (III): F(25%) + O (75%).

### 3.3. Experimental Design

A controlled pot experiment was conducted using the prepared substrates for cucumber seedling production. For this purpose, the prepared substrates were placed in production pots with a capacity of 0.5 L. Then, 3 seeds of the cucumber variety ‘Śremski’ were sown at a depth of 1 cm in each pot. The pots were placed in a greenhouse with an air temperature of ±20 °C and a relative humidity of 80%. The moisture level in the substrate used for *Cucumis sativus* L. seedling production was maintained at 60% of the maximum water-holding capacity (MWHC). The experiment was performed in 3 technological replicas testing six types of compost variants (A, B, C, D, E, F) in four application levels (0%, 0.25%, 0.50%, 0.75%).

After 21 days from the sowing date, a series of measurements and biochemical analyses were performed to determine the impact of the applied substrates on the produced cucumber seedlings.

### 3.4. Analysis of Physicochemical Properties of Substrates

The substrate samples were air-dried and then sieved through a 2 mm diameter mesh. After that, the samples were homogenized. In substrate samples, the following analyses were determined: pH value was determined in a 1:10 substrate–water suspension using a 4221 pH meter (Hanna Instruments, Nusfalau, Romania). Electrical conductivity (EC) was analyzed in a 1:10 substrate–water suspension with a HI 2316 EC-meter from Hanna Instruments (Nusfalau, Romania). The total organic carbon (TOC) and total nitrogen content (N_total_) were determined with the dry combustion method using the Elementar Vario El Cube Analyzer (Elementar, GmbH Germany). The content of Ca, Mg, Zn, Cu, Cr, Ni, Pb, Cd, and Hg in substrate samples was determined by the absorption spectrometric method using the Polarized Zeeman Atomic Absorption Spectrophotometer Hitachi Z-2000 models (Tokyo, Japan) after soil samples mineralization in HNO_3_. Then, the 1 g dry soil samples were weighed in PTFE containers. Afterwards, 10 mL HNO_3_ was added. The microwave system (CEM Mars 5 Microwave Digestion System) was used to prepare samples for Ca, Mg, Zn, Cu, Cr, Ni, Pb, and Cd analysis [42]. The Hg concentration in substrate samples was determined using the HYDRA-C Mercury Analyzer (Teledyne Instruments Leeman Labs Inc. Hudson, New Hampshire, United States). The Egnér–Riehm method has been used for estimating the available phosphorus (P) and potassium (K) [43].

### 3.5. Physiological Parameters of Plants

#### 3.5.1. Relative Chlorophyll Content

The relative chlorophyll content measurement in cotyledons and leaves of cucumber plants was carried out using the chlorophyll content Meter CCM-200plus (Opti-Sciences, Hudson, NH, USA). The measurements were conducted on the 21st day after sowing and were replicated 20 times for each substrate variant..

#### 3.5.2. Chlorophyll Fluorescence

The measurement of selected chlorophyll fluorescence parameters (maximum quantum yield of primary photochemistry, Fv/Fo; maximal photochemical efficiency of PSII, Fv/Fm) in cotyledons and leaves of cucumber seedlings was conducted according to the methodology described in the paper by Matłok et al. [26]. The measurements were taken on the 21st day after sowing and were replicated 20 times for each substrate variant.

### 3.6. Biometric Parameters of Cotyledons and Leaves

The effect of the applied substrates for cucumber seedling production on the size of developed plants was determined through measurements of selected biometric characteristics of cotyledons and leaves. The area and mass of cotyledons and leaves of produced cucumber seedlings were assessed. Measurements of cotyledon and leaf area were conducted using a Leaf Area Meter—AM350 (ADC BioScientific Ltd., Global House, Geddings Road, Hoddesdon, Herts, UK) in 20 replications for each substrate variant. The results were presented in mm^2^. The mass of cotyledons and leaves was determined using a laboratory-scale RadWag WTB200 (RadWag, Radom, Poland). Individual cotyledons and leaves of cucumber seedlings were separated from the rest of the plant and weighed in 20 replications for each substrate variant. The results were presented in grams.

### 3.7. Biochemical Analyzes of Plant Biomass

#### 3.7.1. Enzymes Extraction

The preparation of samples and isolation of analytes from plant material were carried out according to the methodology described by Balawejder et al. [44].

#### 3.7.2. Activity of Superoxide Dismutase, Catalase and Guaiacol Peroxidase

The level of activity of selected enzymes (superoxide dismutase (SOD), catalase (CAT), and guaiacol peroxidase (GPOX)) in the cotyledons and leaves of cucumber plants grown in different substrates was determined according to the methodology described by Balawejder et al. [44].

### 3.8. Statistical Analysis

One-way analysis of variance (ANOVA) was conducted at a significance level of α = 0.05 using STATISTICA 13.1 software (TIBCO Software Inc., Hillview Avenue, Palo Alto, CA, USA). The mean values calculated from the three independent replications were analyzed statistically by comparing the results between the variants of the experiment.

## 4. Conclusions

In summary, while the research has successfully demonstrated the effectiveness of compost and deacidified peat in seedling production, it also highlights the importance of addressing potential stress factors caused by soil organisms. The findings provide valuable insights into optimizing the substrate composition for seedling cultivation and suggest practical applications for the agricultural industry. Further research may focus on mitigating the stress factors associated with these substrates to achieve even better results in seedling production.

## Figures and Tables

**Table 1:** Physicochemical properties of substrates used in the experiment (mean ± SD).

Substrate Variant		Compost Variant + Deacidified Peat (O)	pH	EC	TOC	N_total_	C:N	Available Forms of Nutrients Calculated as Oxides		Total Forms of Nutrients	
								P_2_O_5_	K_2_O	Mg	Ca
		(%)	(H_2_O)	(µS cm^−1^)	(%)	(%)		(mg∙100 g^−1^)		(g∙kg^−1^)	
0	(Control)	O (100%)	6.97 ± 0.21	136 ± 11	55.0 ± 2.6	2.30 ± 0.11	23.9 ± 1.21	46.9 ± 3.5	33.0 ± 2.7	1.37 ± 0.18	2.13 ± 0.11
1	(I)	A (25%) + O (75%)	6.63 ± 0.32	560 ± 24	52.1 ± 3.2	2.73 ± 0.17	19.1 ± 0.97	325 ± 11	123 ± 14	1.83 ± 0.23	5.85 ± 0.18
	(II)	A (50%) + O (50%)	7.05 ± 0.41	774 ± 31	49.1 ± 2.9	3.16 ± 0.19	15.5 ± 0.86	650 ± 34	247 ± 23	2.29 ± 0.17	9.57 ± 0.64
	(III)	A (75%) + O (25%)	6.89 ± 0.37	1134 ± 38	46.2 ± 3.9	3.60 ± 0.09	12.8 ± 0.54	975 ± 45	370 ± 34	2.74 ± 0.21	13.28 ± 0.76
2	(I)	B (25%) + O (75%)	6.85 ± 0.19	570 ± 17	47.5 ± 4.2	2.19 ± 0.13	21.7 ± 0.42	273 ± 27	172 ± 18	1.60 ± 0.16	5.40 ± 0.54
	(II)	B (50%) + O (50%)	6.82 ± 0.25	988 ± 29	40.0 ± 1.9	2.06 ± 0.08	19.4 ± 0.38	546 ± 36	344 ± 26	1.84 ± 0.29	8.67 ± 0.48
	(III)	B (75%) + O (25%)	7.05 ± 0.26	1315 ± 28	32.4 ± 1.6	1.95 ± 0.14	16.6 ± 0.19	818 ± 43	516 ± 34	2.07 ± 0.18	11.93 ± 0.52
3	(I)	C (25%)+ O (75%)	6.91 ± 0.36	556 ± 19	46.8 ± 2.4	1.98 ± 0.17	23.6 ± 0.21	50 ± 2.6	116 ± 12	1.48 ± 0.24	6.47 ± 0.37
	(II)	C (50%)+ O (50%)	7.08 ± 0.29	1104 ± 21	38.5 ± 2.9	1.66 ± 0.12	23.2 ± 0.26	101 ± 12	232 ± 27	1.59 ± 0.16	10.82 ± 0.46
	(III)	C (75%)+ O (25%)	7.28 ± 0.29	1469 ± 32	30.3 ± 1.7	1.34 ± 0.11	22.6 ± 0.27	151 ± 16	347 ± 36	1.69 ± 0.19	15.16 ± 0.57
4	(I)	D (25%)+ O (75%)	6.27 ± 0.17	1398 ± 36	51.8 ± 2.4	2.38 ± 0.28	21.8 ± 0.19	236 ± 32	127 ± 16	1.88 ± 0.11	6.92 ± 0.34
	(II)	D (50%)+ O (50%)	6.09 ± 0.11	1552 ± 27	48.6 ± 2.9	2.45 ± 0.20	19.8 ± 0.25	471 ± 43	255 ± 21	2.39 ± 0.27	11.72 ± 0.47
	(III)	D (75%) + O (25%)	5.90 ± 0.10	1958 ± 32	45.4 ± 3.2	2.52 ± 0.19	18.0 ± 0.34	707 ± 25	382 ± 31	2.89 ± 0.31	16.51 ± 0.57
5	(I)	E (25%) + O (75%)	6.69 ± 0.23	465 ± 18	47.0 ± 2.8	2.20 ± 0.10	21.4 ± 0.19	222 ± 10	191 ± 23	1.78 ± 0.18	5.70 ± 0.32
	(II)	E (50%) + O (50%)	6.90 ± 0.28	1106 ± 24	39.0 ± 2.9	2.08 ± 0.09	18.8 ± 0.18	445 ± 31	382 ± 34	2.19 ± 0.24	9.27 ± 0.31
	(III)	E (75%) + O (25%)	6.59 ± 0.17	1875 ± 29	31.0 ± 2.5	1.98 ± 0.13	15.7 ± 0.23	667 ± 45	572 ± 40	2.59 ± 0.23	12.83 ± 0.48
6	(I)	F (25%) + O (75%)	7.33 ± 0.25	552 ± 17	48.1 ± 3.1	2.12 ± 0.15	22.7 ± 0.31	91 ± 11	182 ± 16	2.30 ± 0.17	7.45 ± 0.47
	(II)	F (50%) + O (50%)	7.46 ± 0.23	863 ± 21	41.2 ± 1.7	1.93 ± 0.17	21.3 ± 0.19	182 ± 17	364 ± 21	3.24 ± 0.32	12.77 ± 0.38
	(III)	F (75%) + O (25%)	7.87 ± 0.31	1680 ± 29	34.3 ± 1.9	1.75 ± 0.23	19.6 ± 0.16	273 ± 23	546 ± 28	4.17 ± 0.29	18.08 ± 0.49

**Table 2:** Content of microelements in substrates used in the experiment (mean ± SD).

Substrate Variant		Compost variant + Deacidified Peat (O)	Pb	Cr	Cu	Ni	Cd	Zn	Hg
		(%)	(mg∙kg^−1^)						
0	(Control)	O (100%)	1.23 ± 0.11	3.45 ± 0.18	12.35 ± 1.12	1.12 ± 0.09	0.05 ± 0.00	127 ± 15	0.01 ± 0.00
1	(I)	A (25%) + O (75%)	3.42 ± 0.12	5.11 ± 0.36	26.76 ± 3.21	2.07 ± 0.76	0.15 ± 0.01	159 ± 19	0.04 ± 0.00
	(II)	A (50%) + O (50%)	5.62 ± 0.09	6.78 ± 0.46	41.18 ± 4.54	3.01 ± 0.43	0.25 ± 0.02	190 ± 21	0.07 ± 0.01
	(III)	A (75%) + O (25%)	7.81 ± 0.24	8.44 ± 0.73	55.59 ± 3.24	3.96 ± 0.23	0.34 ± 0.01	222 ± 32	0.10 ± 0.02
2	(I)	B (25%) + O (75%)	3.42 ± 0.31	5.06 ± 0.68	23.76 ± 2.77	2.04 ± 0.19	0.14 ± 0.00	151 ± 17	0.03 ± 0.00
	(II)	B (50%) + O (50%)	4.57 ± 0.29	6.66 ± 0.54	35.18 ± 3.46	2.96 ± 0.16	0.24 ± 0.01	175 ± 21	0.06 ± 0.01
	(III)	B (75%) + O (25%)	6.23 ± 0.32	8.27 ± 0.67	46.59 ± 5.23	3.88 ± 0.21	0.33 ± 0.03	199 ± 19	0.08 ± 0.01
3	(I)	C (25%)+ O (75%)	2.87 ± 0.16	5.59 ± 0.48	13.26 ± 2.09	2.17 ± 0.11	0.12 ± 0.00	118 ± 10	0.01 ± 0.00
	(II)	C (50%)+ O (50%)	4.52 ± 0.19	7.73 ± 0.38	14.18 ± 1.97	3.21 ± 0.09	0.20 ± 0.01	109 ± 11	0.02 ± 0.00
	(III)	C (75%)+ O (25%)	6.16 ± 0.32	9.86 ± 1.09	15.09 ± 1.34	4.26 ± 0.23	0.27 ± 0.03	100 ± 9	0.02 ± 0.00
4	(I)	D (25%)+ O (75%)	2.82 ± 0.14	5.59 ± 0.83	30.26 ± 3.45	2.04 ± 0.24	0.16 ± 0.03	164 ± 15	0.05 ± 0.00
	(II)	D (50%)+ O (50%)	4.42 ± 0.17	7.73 ± 0.65	48.18 ± 2.78	2.96 ± 0.38	0.28 ± 0.04	201 ± 21	0.10 ± 0.01
	(III)	D (75%) + O (25%)	6.01 ± 0.24	9.86 ± 0.65	66.09 ± 5.67	3.88 ± 0.27	0.39 ± 0.02	237 ± 23	0.14 ± 0.02
5	(I)	E (25%) + O (75%)	2.77 ± 0.16	5.34 ± 0.43	20.51 ± 1.45	1.97 ± 0.12	0.13 ± 0.01	153 ± 24	0.03 ± 0.00
	(II)	E (50%) + O (50%)	4.32 ± 0.27	7.23 ± 0.37	28.68 ± 4.12	2.81 ± 0.32	0.22 ± 0.01	180 ± 19	0.04 ± 0.00
	(III)	E (75%) + O (25%)	5.86 ± 0.31	9.11 ± 0.98	36.84 ± 3.65	3.66 ± 0.31	0.30 ± 0.04	206 ± 21	0.06 ± 0.01
6	(I)	F (25%) + O (75%)	2.87 ± 0.16	5.84 ± 0.56	13.26 ± 2.09	2.12 ± 0.27	0.13 ± 0.01	115 ± 19	0.02 ± 0.00
	(II)	F (50%) + O (50%)	4.52 ± 0.39	8.23 ± 0.62	14.18 ± 2.34	3.11 ± 0.19	0.20 ± 0.02	103 ± 8	0.03 ± 0.00
	(III)	F (75%) + O (25%)	6.16 ± 0.24	10.61 ± 1.04	15.09 ± 1.98	4.11 ± 0.27	0.28 ± 0.04	90 ± 9	0.03 ± 0.00

## Data Availability

Not applicable.
